# Paranasal sinus angiosarcoma with facial paralysis as a novel manifestation: a case report and literature review

**DOI:** 10.1186/s12883-023-03482-2

**Published:** 2023-12-02

**Authors:** Chengcheng Chai, Xiaocong Feng, Kai Li, Zhaoxian Yan, Shuyi Tan, Jin Weng, Fan Huang, Jianpeng Huang, Xinru Zhu, Xuehui Zhuo, Hai Chen

**Affiliations:** 1 The Second Clinical College, Guangzhou University of Chinese Medicine, Airport Road 12, Baiyun District, Guangzhou, Guangdong 510405 China; 2 The Second Affiliated Hospital, Guangzhou University of Chinese Medicine, Dade Road 111, Yuexiu District, Guangzhou, Guangdong 510120 China; 3 Department of Rehabilitation Sciences, The Hong Kong Polytechnic University, Room No. ST512, Hung Hom, Kowloon, Hong Kong Special Administrative Region China

**Keywords:** Paranasal sinus angiosarcoma, Facial paralysis, Pathology, Immunohistochemistry, Case report

## Abstract

**Background:**

Paranasal sinus angiosarcoma is an uncommon malignancy, with only a few reported cases worldwide. Although it exhibits multiple symptoms, facial paralysis has not been previously documented as a noticeable presentation.

**Case Presentation:**

In this case, we report a 40-year-old male who presented with facial numbness and pain for one month, weakness of his facial muscles for 15 days, and recurrent right epistaxis for 1 year. He had a history of nasal inflammatory polyps with chronic sinusitis. Computed tomography and magnetic resonance imaging showed space-occupying lesions in the right nasal cavity and maxillary sinus, with bone destruction occurring in the sinus wall and turbinate. This patient then underwent endoscopic surgery. According to the histopathological and immunohistochemical results, he was eventually diagnosed with paranasal sinus angiosarcoma in April 2021. To date, this patient has not initiated any radiotherapy or chemotherapy and has survived with lymphatic metastasis for at least 3 years.

**Conclusions:**

This manuscript suggests that paranasal sinus angiosarcoma can present with facial paralysis. Moreover, pathological and immunohistochemical tests are still vital for diagnosing paranasal sinus angiosarcoma and differential diagnosis. Additionally, regular follow-up is crucial for patients with paranasal sinus angiosarcoma, enabling monitoring of recurrence, metastasis, and recovery while contributing valuable clinical data to understanding this rare disease and associated research endeavours.

## Introduction

Paranasal sinus angiosarcoma (PSA) is a rare malignant tumour and aggressive soft tissue sarcoma of endothelial origin [1]. Angiosarcomas feature extensive radial invasion in the dermis and have the potential to grow in any soft tissue structure or internal organ of the body [2]. While the trunk is the most common site for patients with a history of cancer, angiosarcomas in the head and neck region are relatively more prevalent among individuals without prior malignancies [3–5]. The modes of dissemination are mainly haematogenous, with most of the metastases taking place in the patient’s lung, but they can also metastases to the bone, liver, soft tissue structure, and lymph nodes [4, 6]. Overall, primary sinonasal tract angiosarcomas account for less than 0.1% of all malignancies in the sinonasal tract region [7]. In general, the overall 5-year survival rate of angiosarcoma is about 12–50% [8, 9]. Risk factors include chronic lymphedema, radiotherapy, vinyl chloride, thorium dioxide, and foreign bodies (such as surgical gauze) [1, 10–12]. A recent project showed that UV damage mutations may be a causative factor of head and neck angiosarcoma [13]. However, the specific etiologies of PSA are still unclear.

Detecting and diagnosing PSA early remains challenging due to the mild initial symptoms. Angiosarcoma occurring in the paranasal sinus can invade surrounding structures, including the lateral nasal wall, anterior wall of the sinus, infraorbital nerves, eyes, and maxilla. This invasion can result in various symptoms, such as epistaxis, nasal congestion, headaches, toothaches, and diplopia [14, 15]. Previously, it has been reported that angiosarcomas of the head and neck may cause facial sensory abnormalities [15], but facial paralysis (FP) as an obvious symptom of PSA was hardly mentioned.

Here, we report a case of PSA with unilateral FP as a novel manifestation. The severity of FP was assessed by the House-Brackman (H-B) grading system. We discuss the relationship between FP and PSA, analyse the differential diagnosis of PSA from pathological and immunohistochemical perspectives, and draw valuable lessons from this case.

## Case presentation

**Fig. 1:**
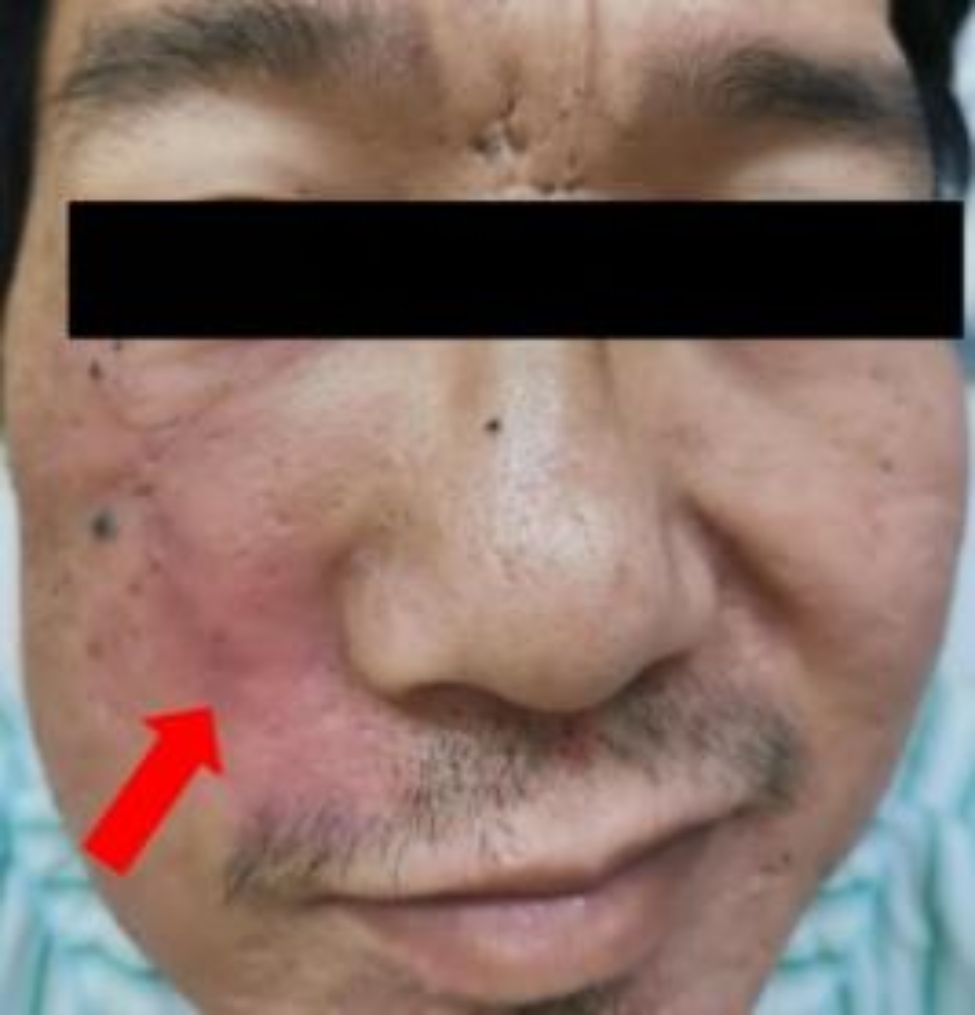
The symptom of facial paralysis in this patient

**Fig. 2:**
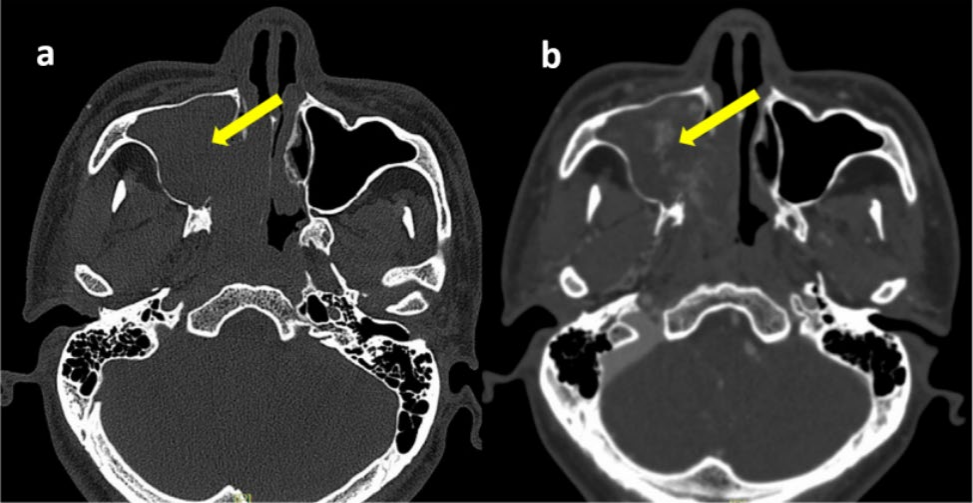
CT scan of the paranasal sinus (2020-01-08) (**a**). Enhanced CT scan of the paranasal sinus (2020-01-15) (**b**). The tumour occupied part of the right maxillary sinus and nasal cavity (arrow)

In April 2021, a 40-year-old man came to the Guangdong Provincial Hospital of Chinese Medicine ENT department complaining of facial numbness and pain for one month, weakness of his facial muscles for 15 days, and recurrent right epistaxis for 1 year. Initially, he experienced numbness and pain on the right side of his face, followed by abnormal flushing on the right midface approximately 10 days later. He developed sagging at the corners of the right side of his mouth and a deepening of the right nasolabial groove (Fig. 1). Of note, he had a smoking history of over 20 years and a medical history of right nasal inflammatory polyps with chronic sinusitis for more than 1 year. Meanwhile, he denied a history of occupational exposure and other associated nasal diseases. In 2020, he presented with right epistaxis and nasal obstruction for over 1 year. Computed tomography (CT) revealed that the tumour occupied part of the right maxillary sinus and nasal cavity (Fig. 2a and b). He underwent his first functional endoscopic sinus surgery (FESS) in January 2020. However, this surgery was aborted due to excessive bleeding (more than 2000 millilitres), leading to only partial mass removal. Some tissue in the right paranasal sinus could not be removed and sent for pathological examination. Histopathology results of the removed masses confirmed the diagnosis of nasal inflammation polyps with massive necrosis due to the patient’s refusal to undergo further immunohistochemical examination of this portion of tissue, which led to difficulties in determining the nature of the tumour. He was preliminary diagnosed with right nasal inflammatory polyps and chronic sinusitis and discharged. Then, appropriate intranasal treatment was administered, including a 0.9% sodium chloride injection to rinse the nasal cavity, oral antihistamines, mucosal excretion enhancers, and a full course of antibiotics. After receiving the above-mentioned intranasal treatments for a while, the patient felt that his nasal obstruction symptoms had been alleviated. At that time, he discontinued them on his own and never received similar treatment again. However, he had developed symptoms of right epistaxis again in April 2020.

Clinical examination revealed tenderness in the right ethmoid sinus and maxilla, as well as dense secretions in the right middle nasal meatus. Additionally, hypoesthesia was observed in the maxillary nerve distribution area, as well as in the distribution areas of the zygomatic and buccal branches of the right facial nerve, accompanied by the loss of the ability to whistle. These findings indicated FP (H-B III).

**Fig. 3:**
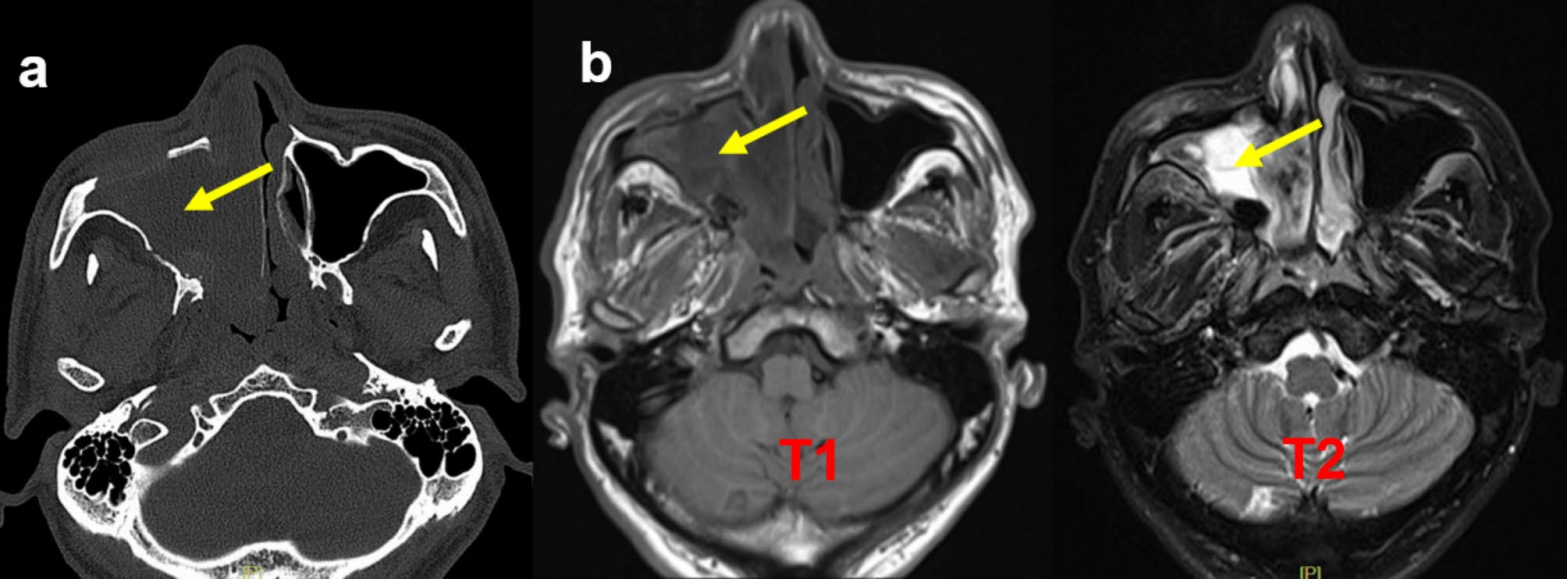
CT scan of the paranasal sinus (2021-04-15) (**a**). Enhanced MRI Scan of the paranasal sinus (2021-04-20) (**b**). The mass in the right maxillary sinus had by the sinus (arrow)

CT and Magnetic Resonance imaging (MRI) revealed an increased extent of the space-occupying lesions in the right nasal cavity and maxillary sinus compared to the images from 2020 (Fig. 3a and b). From the presented data, it was evident that the mass in the right maxillary sinus had occupied entirely the sinus, ruptured it, and obstructed the right common nasal meatus and middle nasal meatus. Moreover, the anterior wall of the right maxillary sinus had also been damaged. Although the intranasal biopsy results pointed towards necrotising polyps, the recurrence of the lesion indicated the possibility of malignant changes. However, the doctor decided to perform a second FESS. During this procedure, a significant amount of red and grey tissue was extracted from the right maxillary sinus, and extensive bone destruction was observed. The tumour was excised and sent for pathological testing.

**Fig. 4:**
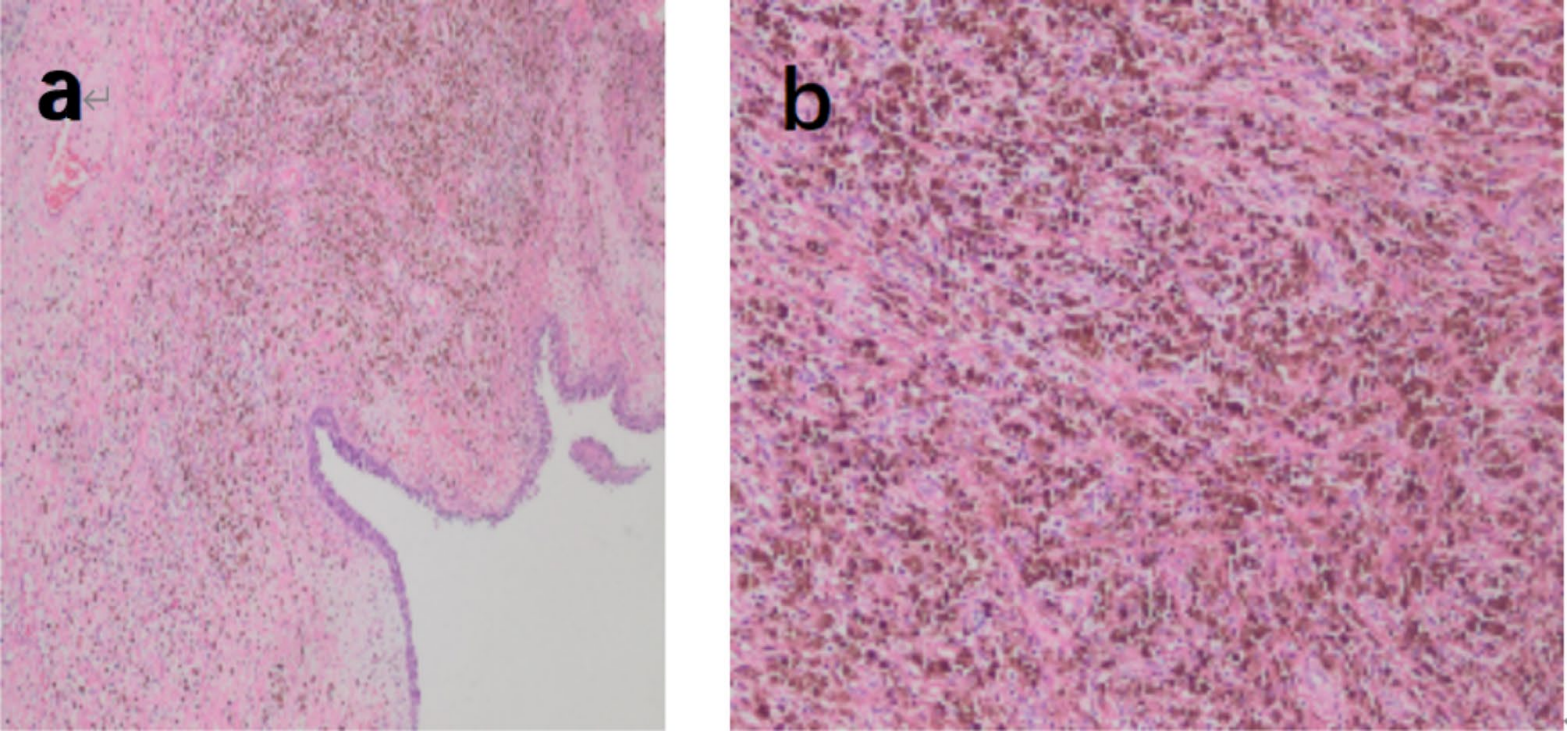
Pathological result: The presence of more adipose spindle cell hyperplasia under the mucosa of the right maxillary sinus mass and the mitotic image (X200) (**a**). Hematoxylin-eosin staining revealed angiosarcoma (X400) (**b**)

**Fig. 5:**
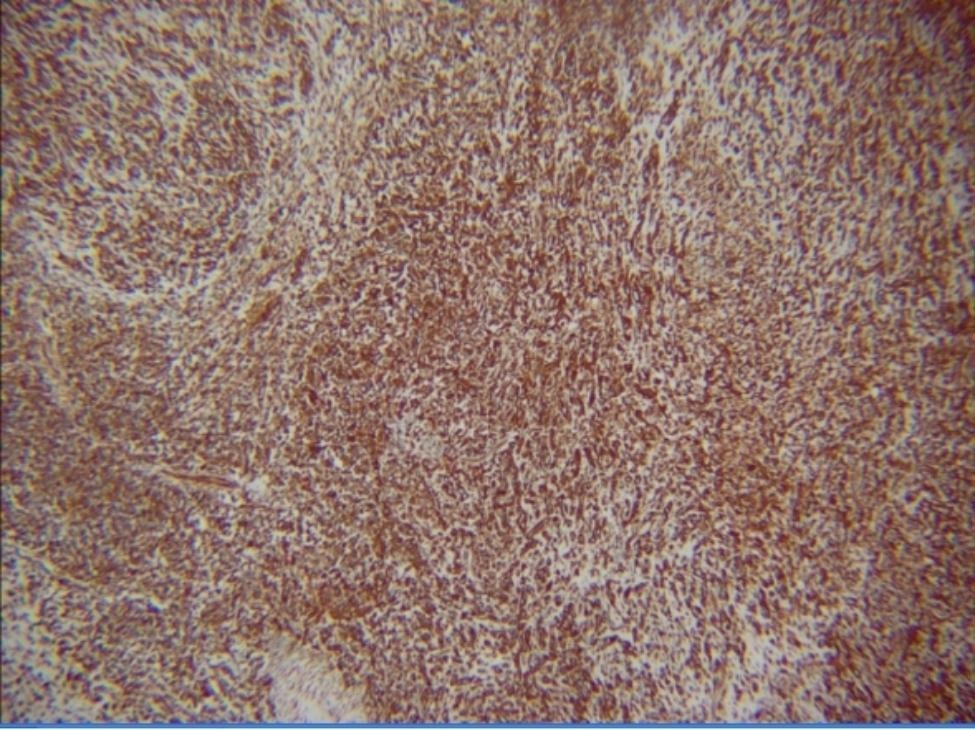
Immunohistochemical result: The proliferation index, expressed as a percentage of Ki-67 antigen-positive nuclei, was approximately 30% of cells. The results also suggested: P16 (+), CD31 (+), and ERG (+)

The postoperative pathological result revealed the presence of more adipose spindle cell hyperplasia under the mucosa of the right maxillary sinus mass, and the findings of mitotic figures on the images (Fig. 4a and b) suggested a malignant lesion. Eventually, the patient was diagnosed with PSA according to the result of immunohistochemistry, which suggested Ki67 (approximately 30% +, P16 (+), CD31 (+), and ERG (+) (Fig. 5). According to the eighth edition of American Joint Committee on Cancer (AJCC) cancer staging manual [16], the patient was classified as stage T4aN2aM0. Despite our careful explanation of the potential consequences and prognosis, the patient and his family rejected the subsequent treatment: local extended resection combined with radiotherapy and chemotherapy.

**Fig. 6:**
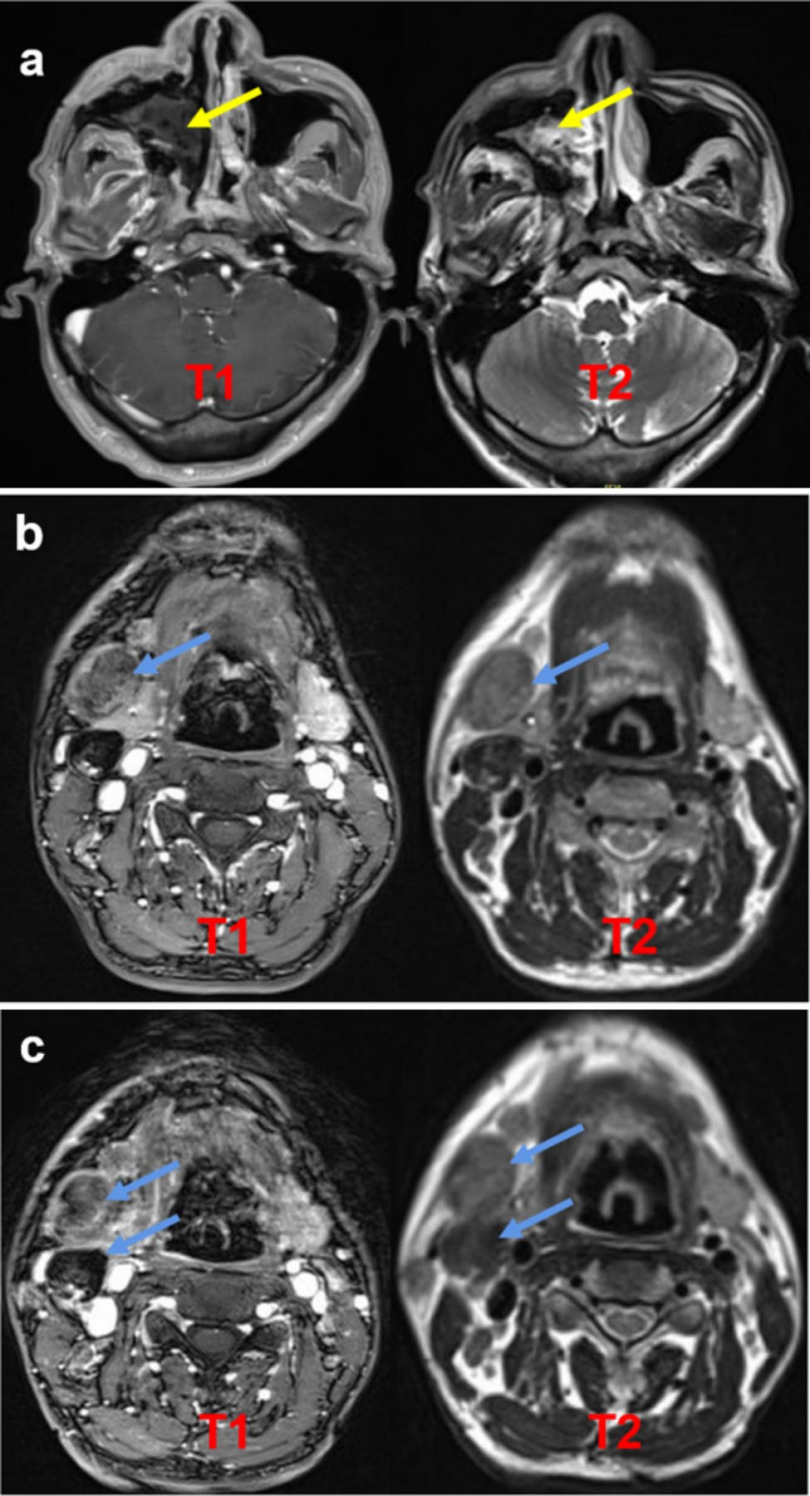
Enhanced MRI scan of the paranasal sinus (2021-08-20). The tumour had reoccupied the right maxillary sinus and the right common nasal meatus (arrow) (**a**). Enhanced MRI scan of the neck (2021-08-20). Lymphatic metastasis had occurred in the right neck, and the largest lymph nodes were located in the IB area of the right neck (2.8 cm × 2.0 cm) (arrow) (**b**). Enhanced MRI scan of the paranasal sinus and neck (2021-12-02). The space-occupying area in the right maxillary sinus had decreased in size, while the IB area of the right neck (2.3 cm × 3.9 cm) was larger than before, with multiple lymph nodes present in the bilateral neck IV region (arrow) (**c**)

At the 3-year follow-up, the patient described alleviating his facial numbness and hypoesthesia following surgery. Additionally, after being diagnosed with PSA, he chose to quit smoking. However, in August 2021, MRI scans of the sinus and neck revealed that the tumour may have infiltrated the skin along the bone defect of the anterior wall of the right maxillary sinus (Fig. 6a). Concurrently, lymphatic metastasis had occurred in the right neck, with the largest lymph nodes located in the IB area of the right neck (2.8 cm × 2.0 cm) (Fig. 6b). In December 2021, MRI indicated a reduction in the size of the space-occupying area in the right maxillary sinus, while the IB area of the right neck (2.3 cm × 3.9 cm) was larger than before, with multiple lymph nodes present in the bilateral neck IV region (Fig. 6c). Based on these data, the stage of this patient was updated as T4aN2bMx.

**Fig. 7:**
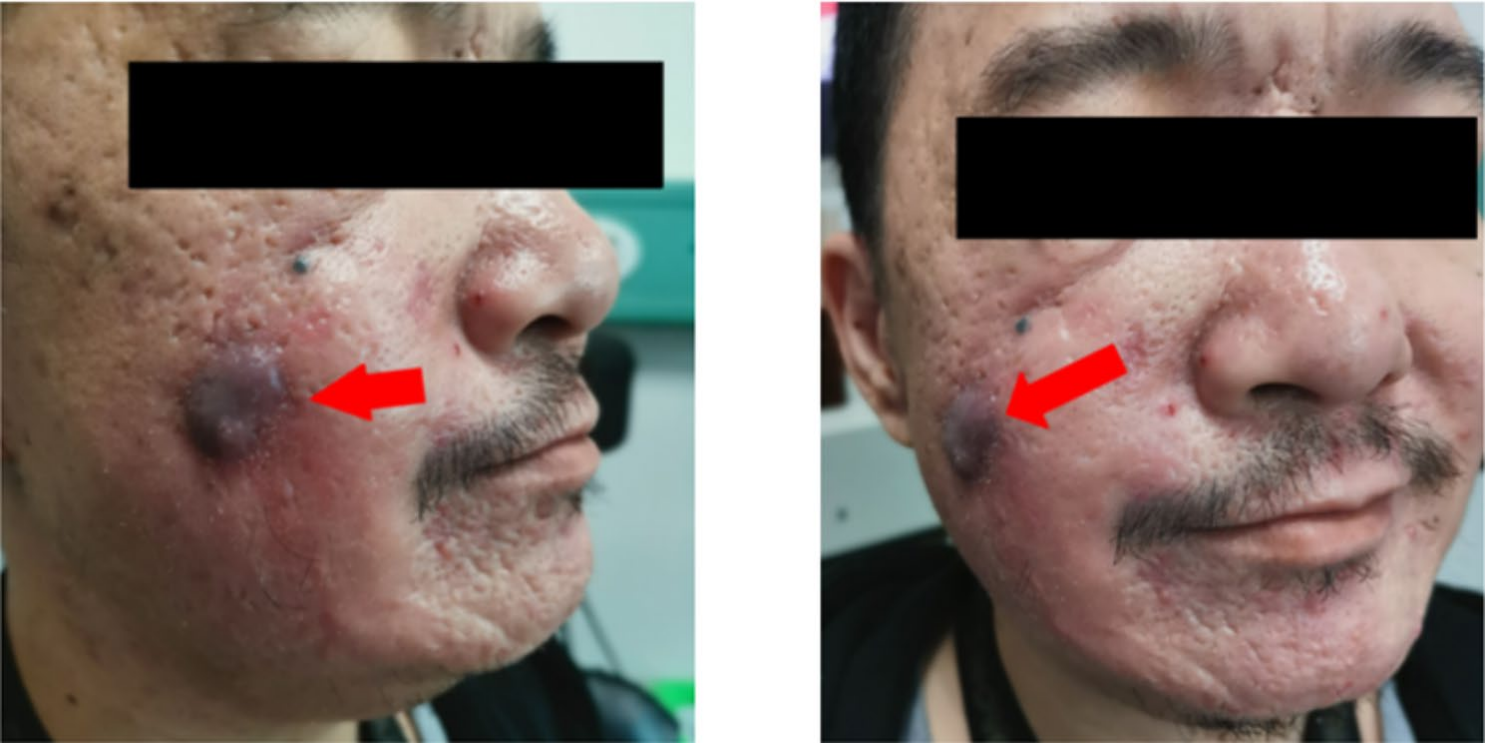
The nodule grew on the right face of the patient

In November 2022, the patient developed a purplish-red induration on his cheek (Fig. 7), but the severity of FP was not increased. Unfortunately, the patient declined to undergo nasal endoscopy, biopsy, CT, and MRI examinations, which made it challenging to determine the progression of the lesion in the right maxillary sinus and nasal cavity and whether this nodule was consistent with PSA. Despite experiencing recurrent epistaxis and impaired facial function, he has survived for more than 3 years without receiving any radiotherapy or chemotherapy since being diagnosed with PSA.

## Discussion

Although the causes of FP are various, the influence of tumours cannot be ignored. In a series of 300 patients with treatable FP seen by otolaryngologists, 112 of these patients were confirmed to have a tumour as a pathogenesis of FP [17]. However, FP is an uncommon symptom of angiosarcoma. We reviewed the previous literature and found that only 2 cases of angiosarcoma were associated with FP. One case involved metastasis of primary cardiac angiosarcoma to the meninges [18], and the other case described angiosarcoma growth in the temporal bone [19]. In patients with head and neck cancer, FP can result in detrimental facial function and mental health, compounded by the incidence rate linked to the patient’s oncologic illness and treatment [20–22]. In our case, the patient’s nerve injuries mostly involved the maxillary nerve, right zygomatic branches, and buccal branches, which affected his appearance and basic daily activities (including eating, drinking, and so on).

To our knowledge, perineural invasion (PNI) and perineural tumour spread (PNTS) are well-documented modes of dissemination for head and neck malignancies [23]. Symptoms related to the nerves (such as FP and trigeminal neuralgia) resulting from PNI and PNTS from malignancy are relatively common in the literature [24–26]. However, in this case, PNI and PNTS are both unlikely to be associated with FP. PNI has been demonstrated as a common pathologic finding in head and neck malignancies, especially in squamous cell carcinoma (25–80%) and adenoid cystic carcinoma (at least 50%) [27–29], while it has not yet been observed in angiosarcoma. Additionally, the typical pathological patterns of PNI include the following: i) tumour cells are close to the nerve and involve at least 33% of the surrounding area and ii) tumour cells are located in the perineural nerve sheath and can differentially infiltrate into the three nerve sheaths [30]. We did not find direct evidence of such patterns in the patient’s pathological results. Although a previous case report revealed that angiosarcoma has the possibility of PNTS [31], we have not yet discovered conclusive evidence of PNTS in our patient’s MRI scans, which are the most sensitive tool for detecting PNTS [25]. However, as an aggressive tumour, the potential of PI and PNTS in PSA cannot completely be ruled out.

The connection between FP and PSA may be interpreted by the anatomy of the paranasal sinuses, trigeminal nerve, and facial nerve. The maxillary nerve, as one of the second branches of the trigeminal nerve, plays a significant role in supplying sensory components to the nasal cavity and paranasal sinuses [32]. In addition, the paranasal sinus are also one of the mucous areas of the maxillary nerve [32]. Furthermore, the maxillary nerve has the highest frequency of communication with the facial nerve [33]. Among the various terminal branches of the facial nerve, the zygomatic branches and buccal branches have the most frequent communication [34]. Furthermore, previous studies have established that solid tumours require a blood supply to grow [35]. Therefore, as angiosarcoma grows, it can oppress the nerves around the paranasal sinus and steal the blood supply that is supposed to belong to the nerve, which ultimately leads to FP. The patient’s nerve damage may first occur in the maxillary nerve and then involve the zygomatic branches and buccal branches, consistent with the order of his symptoms.

During the initial operation, the masses were diagnosed as inflammatory polyps of the nasal cavity and paranasal sinuses. However, considering the patient’s medical history, we suspected that the masses may not have been solely inflammatory polyps. We thought they might have been tumours that exhibited abnormal differentiation due to unknown factors during the process of a recurrence. Unfortunately, our speculation could not be confirmed at the time due to limitations in medical techniques. Additionally, some antigens were lost in the pathological specimen over time, which hindered the immunohistochemical tests. This dilemma highlights the importance of timely pathological and immunohistochemical tests when diagnosing accurately and distinguishing it from other diseases.

PSA should be differentiated from reticulohistocytoma, intravascular papillary endothelial hyperplasia, epithelioid sarcomas, and melanoma. Table 1 shows the similarities and differences between PSA and these diseases.

**Table 1:** Similarities and differences between paranasal sinus angiosarcoma and other diseases

Diseases	Similarities	Differences
Reticulohistocytoma	• Infiltrate dermis • CD31(+) Vimentin (+) Human Melanoma Black-45(-) [36]	• Angiosarcomas: CD34 (+), Keratin (+), S-100 (-), Lysozyme (-), CD68 (-) • Angiosarcomas often invade contiguous bone, soft tissues, and cartilage, as seen on a CT scan • Angiosarcomas have haemorrhagic infiltration [37]
Intravascular papillary endothelial hyperplasia	• Predominant endothelial cell hypertrophy and formation of multiple papillae • Bone erosion on CT scan • CD31(+) CD34(+) Vimentin (+) [38]	• Angiosarcomas are invasive and rarely intravascular • Angiosarcomas have a lot of necrotic and solid areas • Angiosarcomas have pleomorphic cellularity and abundant mitotic morphology [38–40]
Epithelioid sarcoma	• Pleomorphism, spindle cells • cutaneous lesions • CD34(+) ETS-related gene (+) [41, 42]	• Angiosarcomas express CD31, and no loss of INI/SMARCB [1] • The pleomorphism of epithelioid sarcoma is usually mild to moderate [43]
melanoma	• Erythrophagocytosis • Spindled, epithelioid, or plasmacytoid • Large and prominent nucleoli [43] • Cytoplasmic hemosiderin pigment	• Angiosarcomas express vascular markers and are negative for S100, Sox10, Melan-An, Human Melanoma Black-45 [43]

There is currently no convincing conclusion regarding the optimal management of PSA. Surgical resection is still the primary therapeutic for angiosarcomas at present [1]. When devising the surgical strategy, it is advisable to maximise the expansion of the incisal margin. The utilisation of endoscopic surgery may be a viable alternative to be taken into account; however, it is crucial to anticipate the resection of the safe margin of the soft tissue/bone ban [44]. In cases when endoscopic surgery fails to ensure complete tumour excision while maintaining an adequate safety margin, alternative approaches such as open surgery should be considered. Besides, PSA is an extremely vascular tumour, so surgeries may become an excess exsanguinating event whenever surgeries aim to diagnose or treat [45]. This case also highlights the importance of this lesson. In addition, the risks and challenges associated with operations are also increased due to the tumour location, the relationship between the tumour and other anatomical tissues, and the multifocal nature of the tumour [1, 46].

Studies have shown that the benefit of radiotherapy and chemotherapy in this disease is indistinctive, but targeted agents and immunotherapy show promise [47, 48]. For instance, one study demonstrated that patients with nasal angiosarcoma may benefit from the vascular endothelial growth factor-A monoclonal antibody, bevacizumab [49]. However, the effectiveness of these treatment approaches needs to be supported by more evidence.

## Conclusions

This case alerts otorhinolaryngologists that although PSA with FP as the symptom is rare in clinical practice, the possibility of PSA should be considered when common causes are excluded, especially in patients with unilateral recurrent epistaxis. ENT doctors need to be fully aware of the risks of facial nerve damage and massive bleeding when formulating a surgical plan for PSA. Moreover, the importance of pathological and immunohistochemical tests in diagnosing PSA should be emphasized.

## Data Availability

All data generated or analyzed during this study have been included in this article.

## Abbreviations

CT: Computed Tomography
FESS: Functional Endoscopic Sinus Surgery
FP: Facial Paralysis
MRI: Magnetic Resonance Imaging
H-B: House-Brackman
PNI: Perineural Invasion
PSA: Paranasal Sinus Angiosarcoma
PNTS: Perineural Tumor Spread
